# Melanoma cell therapy: Endothelial progenitor cells as shuttle of the MMP12 uPAR-degrading enzyme

**DOI:** 10.18632/oncotarget.1987

**Published:** 2014-05-19

**Authors:** Anna Laurenzana, Alessio Biagioni, Silvia D'Alessio, Francesca Bianchini, Anastasia Chillà, Francesca Margheri, Cristina Luciani, Benedetta Mazzanti, Nicola Pimpinelli, Eugenio Torre, Silvio Danese, Lido Calorini, Mario Del Rosso, Gabriella Fibbi

**Affiliations:** ^1^ Department of Experimental and Clinical Biomedical Science, University of Florence, Italy; ^2^ IBD Center, Humanitas Clinical and Research Center Rozzano (Mi), Italy; ^3^ Cord Blood Bank, Careggi University Hospital, Florence, Italy; ^4^ Clinical, Preventive and Oncologic Dermatology Section, Department of Surgery and Translational Medicine, University of Florence, Italy; ^5^ ITT, Istituto Toscano Tumori

**Keywords:** uPAR, MMP12, Endothelial Progenitor Cells, melanoma, cell-therapy

## Abstract

The receptor for the urokinase-type plasminogen activator (uPAR) accounts for many features of cancer progression, and is therefore considered a target for anti-tumoral therapy. Only full length uPAR mediates tumor progression. Matrix-metallo-proteinase-12 (MMP12)-dependent uPAR cleavage results into the loss of invasion properties and angiogenesis. MMP12 can be employed in the field of “targeted therapies” as a biological drug to be delivered directly in patient's tumor mass. Endothelial Progenitor Cells (EPCs) are selectively recruited within the tumor and could be used as cellular vehicles for delivering anti-cancer molecules. The aim of our study is to inhibit cancer progression by engeneering ECFCs, a subset of EPC, with a lentivirus encoding the anti-tumor uPAR-degrading enzyme MMP12. Ex vivo manipulated ECFCs lost the capacity to perform capillary morphogenesis and acquired the anti-tumor and anti-angiogenetic activity. In vivo MMP12-engineered ECFCs cleaved uPAR within the tumor mass and strongly inhibited tumor growth, tumor angiogenesis and development of lung metastasis.

The possibility to exploit tumor homing and activity of autologous MMP12-engineered ECFCs represents a novel way to combat melanoma by a “personalized therapy”, without rejection risk.

The i.v. injection of radiolabelled MMP12-ECFCs can thus provide a new theranostic approach to control melanoma progression and metastasis.

## INTRODUCTION

Many data of the last decades have demonstrated that the urokinase plasminogen activator system (urokinase-type plasminogen activator, uPA; uPA receptor, uPAR; uPA inhibitor type-1, PAI-1) plays a central role in the progression, metastasis, angiogenesis and stemness of numerous solid tumors [1-4]. Endogenous levels of uPA and uPAR increase with disease progression, correlate with poor prognosis and therefore can be considered a good target for anti-tumoral therapy [5]. This membrane-associated system has reached the higher level-of-evidence (level-1) ever for any biomarker in node-positive breast cancer and is actually considered the main system involved in tumor invasion and metastasis, as well as in tumor angiogenesis [6]. uPAR is critical in invasive properties of malignant cells since it is involved both in extracellular matrix (ECM) degradation and in cell adhesion through a “grip-and-go” mechanism [7]. uPAR consists of 3 globular domains (D1, D2, D3) and is present on the cell membrane both as a full-length and a truncated molecule (D2+D3) which lacks the external uPA-binding D1 domain. However, only full length uPAR is able to mediate uPAR-dependent angiogenesis and invasion of cancer cells. In fact, MMP12-dependent uPAR cleavage impairs cell invasivity and angiogenesis [8-9] and uPAR truncation by transient transfection of *MMP12* in a sub-type of EPC, termed “Endothelial Colony Forming Cells” (ECFCs), resulted in inhibition of angiogenesis *in vitro* and *in vivo* [10].

MMP12 is a metalloelastase first identified as a protein secreted by macrophages [11]. MMP-12 shares many features typical of MMPs including the capacity to hydrolyze some extracellular matrix components [12]. Even so, while MMPs commonly facilitate tumor progression, MMP12 displays a controversial role in cancer progression [13]. In fact, despite evidences about correlation of tumor MMP12 with poor prognosis in several tumors [14-16], there are growing evidences about the “protective role” of MMP12, in tumor progression. Notably, overexpression of MMP-12 is associated with reduced tumor growth rates in mice, leading to a favourable outcome [17]. Some authors showed that the effect of MMP12 is determined by cell-type expression: when expressed by host macrophages, it has a protective effect, while when expressed by tumor cells it did not [18]. These observations may possibly account for the failure of clinical trials which rely on the use of broad-range MMP inhibitors [19-20]. Taken together these data indicate that the role of MMP12 in human cancer is still much discussed and that it depends on its specific protein target and cleavage products. The anti-tumor and anti-angiogenetic activity of MMP-12 is often ascribed to the generation of angiostatin from plasminogen [21-22]. Another potential target of MMP12 is uPAR, which can be cleaved, thus abolishing uPA-induced endothelial cell proliferation [23]. On these basis and on the above mentioned results obtained in our laboratory [7-9], MMP12 could be considered and used as a biological drug thus opening a new anti-tumoral therapy focused on uPAR cleavage. In this study we evaluated the possibility to deliver MMP12 into tumor mass where it could cleave uPAR of tumor cells and ECs.

Many studies suggest a role of Endothelial Progenitor Cells (EPCs) in tumor vascularization and metastasis [24]. EPCs are selectively recruited within the tumor mass [25], the amount of circulating EPCs positively correlates with tumor progression and their levels decrease after chemotherapy [26]. Also, Mesenchymal stem cells (MSCs) are able to support tumor angiogenesis and tumor development by providing the matrix required for new vessels and tumor cells scaffolding [27-28]. Due to their capability to home within tumors, EPC and MSC can be proposed to be used in the anti-cancer “cell therapy” as cellular vehicles for delivering molecules inhibiting cancer progression.

The aim of our study was directed to control the progression of those tumor which heavily depend on uPAR to perform invasion and metastasis. As tumor model we utilized melanoma cell lines derived from patients with cutaneous melanoma.

Here we used MSCs as tumoral promoters by co-injecting them in mice together with cancer cells to favour tumor development and showed that MSCs promoted cancer development. On the other side, ECFCs, engineered with a lentivirus encoding MMP12, have been used as carriers of the anti-tumor uPAR-degrading enzyme. We first demonstrated that intravenous injected ^111^In-oxine labelled ECFCs are able to home into tumor mass by exploiting the CXCR4/SDF1 axis. We also demonstrated that the i.v. injected MMP12- engineered “commandos ECFCs” were able to control melanoma progression, angiogenesis and metastasis and, at the same time, to cleave uPAR on tumor cells and endothelial cells of the tumor microenvironment in human melanomas transplanted in nude mice. Our results show that administration of autologous ^111^In-oxine labelled MMP12-lentiviral modified ECFCs can provide a theranostic appoach to human melanoma progression and metastasis.

## RESULTS

### Role of tumor microenvironment on full length uPAR-dependent invasivity of melanoma cells

**Figure 1:**
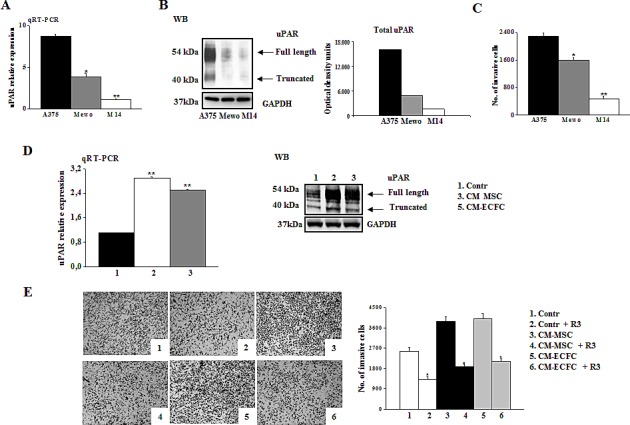
uPAR regulates invasion of melanoma cells Panel A: uPAR expression in A375, Mewo and M14 melanoma cells determined by qRT-PCR. Results are normalized to the expression of ribosomal 18s. Panel B: Western blotting detection of full-length and truncated form of uPAR in melanoma cells. Three experiments were performed for each cell line. GAPDH expression functions as a control for protein loading. Panel C: Matrigel invasion of A375, Mewo and M14 under control conditions. In all invasion assays cells migrating through the Matrigel on the filters of Boyden chambers were counted after 6h and expressed as the total cell number of invading cells/filter. Data presented in panel A and C result from 3 independent experiments expressed as means ± SD. * *P*<0.05 or ** *P*<0.001, with respect to A375 melanoma cells. Panel D: uPAR expression, evaluated by qRT-PCR and by Western blotting in A375 melanoma cells in control condition and after overnight incubation with conditioned medium of MSC (CM-MSC) and ECFC (CM-ECFC). Data result from three independent experiments ± SD. *Asterisks indicate* significant difference (** *P*<0.001) with respect to control. Panel E: Pictures shown here represent Matrigel-coated filters of a typical invasion experiment of A375 melanoma cells in control condition and after stimulation with CM-MSC or CM-ECFC, in the absence and in the presence of anti-uPAR antibody R3. Four experiments were performed for each experimental condition. Histogram on the right shows the quantification performed as in C. *Asterisks* (* *P*<0.05) indicate significant difference between the indicated experimental condition.

**Figure 2:**
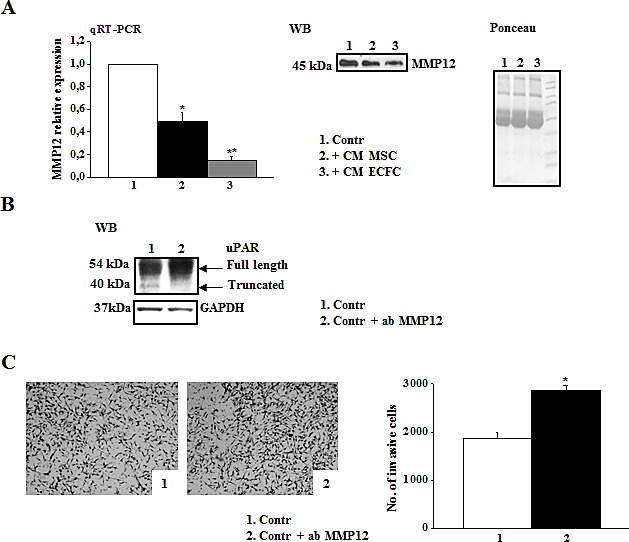
MMP-12 down-regulation correlates with high levels of full length uPAR and A375 invasiveness Panel A: MMP12 levels of A375 were examined by qRT-PCR in control condition or after overnight treatment with CM-MSC or CM-ECFC. Results are normalized to control (assumed as value 1). *Asterisks indicate* significant difference (* *P*<0.05; ** *P*<0.001) from control. MMP12 released in the medium by the same cells was evaluated by Western blotting (on the right). The result is representative of three separate experiments. Ponceau on the right shows equal loading. Panel B: Western blotting analysis of uPAR expressed by A375 cells in control condition and in the presence of anti-MMP12 antibody. Result is representative of three independent experiments. Panel C: Pictures represent the Matrigel-coated filters of an invasion experiment of A375 cells in the same experimental condition described above (panel B). Histogram shows the quantitative analysis of Matrigel invasion. *Asterisks indicate* significant difference (* *P*<0.05) from control.

**Figure 3:**
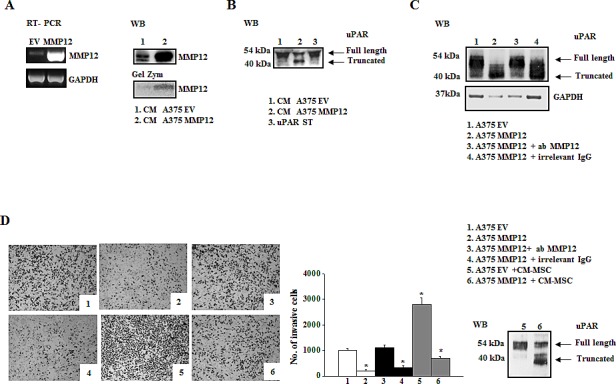
MMP12 inhibits uPAR-dependent invasion of A375 melanoma cells Panel A: transient transfection of A375 cells with the recombinant vector *pCDNA3.1 + MMP12* (MMP12) or empty vector *pCDNA3.1* (EV) as control. RT-PCR analysis of MMP12 in transfected cells (on the left) and Western blotting analysis of MMP12 released in the medium by the same cells (on the right). The enzymatic activity of MMP12 was revealed by the zymographic assay of CM from transiently transfected cells on α-elastin gel (low on the right). Panel B: Western blotting analysis of standard uPAR (st) incubated with aliquots of the culture medium EV (CM-A375-EV) and MMP12 A375 cells (CM-A375-MMP12). Panel C: Western blotting analysis of uPAR expression in A375-EV (mainly full length uPAR) and A375-MMP12 cells (truncated uPAR) in the absence and in the presence of anti-MMP12 antibody or irrelevant IgG. All the images in panels A-C are representative of 3 different experiments that gave similar results; Panel D: Representative fields of invasive transiently transfected A375 cells (empty vector and MMP12), in the presence or absence of anti-MMP12 antibody or of an irrelevant IgG (picture 1-4). Pictures 5 and 6 show the Matrigel invasion of control A375 cells and of MMP12 transfected cells incubated with CM-MSC. The bar chart represents the relative quantification of Matrigel invasion performed by counting migrated cells as described in Figures 1A. Results show data from three independent experiments performed in triplicate. *Asterisks* indicate significant difference from control (A375-EV) (* *P*<0.05;). Full length and truncated form of uPAR in lysates of A375 cells in the conditions shown in pictures 5 and 6 of panel D, was evaluated by Western blotting using R4 anti-uPAR antibody.

We evaluated the correlation between uPAR levels and the invasive properties of three different melanoma cell lines (M14, Mewo, and A375). A375 cells displayed higher uPAR mRNA (Fig.1A) and protein levels, as demonstrated by Western blotting analysis and densitometric evaluation (Fig.1B) as well as higher invasion activity compared to the other cell lines (Fig.1C). In all the cell lines uPAR is mainly present in its biological full length form and only a minor part is cleaved. On the basis of these results, we chose A375 melanoma cells to perform the following experiments. The invasive property of melanoma cells is mainly uPAR-dependent and it is inhibited in the presence of anti-uPAR R3 antibody, as demonstrated in fig 1E, pictures 1 and 2. To investigate the influence of the tumor microenvironment on uPAR expression and invasiveness of A375 melanoma cells we incubated melanoma cells with CM-MSC or CM-ECFC and we observed an increased uPAR expression both at mRNA and protein levels (Fig.1D). Western blotting analysis with the anti-uPAR R4 antibody, which recognizes both full length and truncated uPAR, demonstrated that the molecular form of upregulated uPAR is mainly the native D1+D2+D3 molecule (Fig.1D). Moreover, CMs treatment increased invasive properties of melanoma cells in an uPAR-dependent manner (Fig.1E), since CM-induced invasion was impaired by anti-uPAR R3 antibody. We previously demonstrated that the full length uPAR is required to perform invasion and that uPAR cleavage by the matrix metalloproteinase 12 (MMP12) strongly decreased uPAR-dependent invasion of tumor cells and endothelial cells [8-10]. Therefore, we investigated whether CMs treatment affected MMP12 synthesis and, consequently, uPAR truncation. CM-MSC and CM-ECFC treatment induced MMP12 down regulation both at mRNA and protein level (Fig.2A), which could account for the CM-dependent up-regulation of the full length form of uPAR (Fig.1D) and the increased invasiveness (Fig.1E) of A375 cells. In agreement with these observations, treatment of A375 with MMP12 blocking antibody resulted in the disappearance of uPAR cleaved fragment and enhanced full-length uPAR (Fig.2B) as well as A375 invasiveness (Fig 2C). We observed the same results also with CM-treated A375 cells, in which the treatment with anti-MMP12 antibody induced up-regulation of full length uPAR and the increased invasion (data not shown). Afterwards, we transiently transfected A375 melanoma cells with a MMP12 cDNA containing vector (A375-MMP12) and analyzed their biological characteristics with respect to empty vector transfected cells (A375-EV). MMP12 transfection was effective in upregulating MMP12 mRNA levels and in increasing the release of active protein into the culture medium as revealed by Western blotting analysis and by elastin zymography (Fig.3A). Moreover, we demonstrated that released MMP12 was able to cleave uPAR: by incubating standard uPAR with CM from EV transfected cells (CM-A375-EV) or from MMP12 tranfected cells (CM-A375-MMP12) only CM-A375-MMP12 truncated standard uPAR (Fig.3B). At the same time transfected A375 expressed the truncated form of uPAR on their surface, revealed by Western blotting of cell lysates with the specific anti-uPAR R4 antibody (Fig.3C). Moreover, the invasive property of A375-MMP12, exhibiting cleaved uPAR, was very low as compared to Matrigel invasion of A375-EV, which displayed full length uPAR (Fig.3D). To further evaluate whether MMP12-mediated uPAR cleavage is responsible for inhibition of cell invasion, we neutralized MMP12 in A375-MMP12 cells by using anti-MMP12 antibody. In this experimental conditions melanoma cells expressed full length uPAR (fig. 3C) and, at the same time, the invasion property was completely recovered (fig.3D). On the other hand, in the presence of irrelevant IgG A375-MMP12 cells expressed truncated uPAR and displayed a decreased Matrigel invasion.

Notably, MMP12-transfected A375 cells were unable to respond to CM-MSC stimulation in terms of cell migration when compared to A375-EV. This effect was due to the release of active MMP12 by transfected cells, which cleaved CM-MSC-up-regulated uPAR, as showed by Western blotting of cell lysates (Fig. 3D, pictures 5 and 6).

### Role of SDF1-CXCR4 system in *in vitro* recruitment of microenvironmental cells (ECFC, MSC)

**Figure 4:**
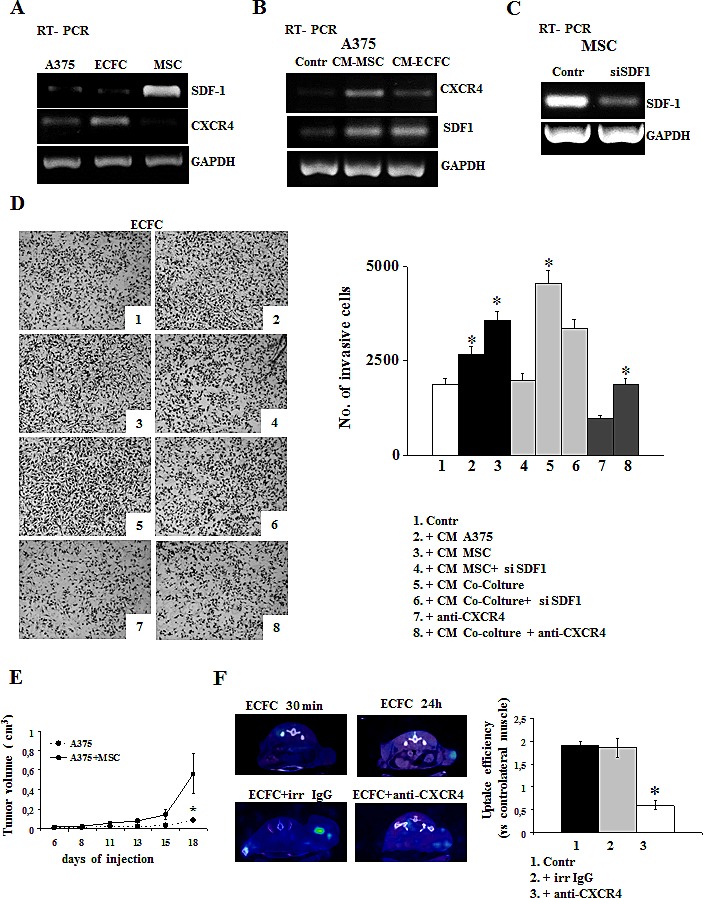
The Stromal cell-derived factor-1/CXCR4 system promotes ECFC recruitment in *in vitro* and *in vivo* Expression of CXCR4/SDF1 system in A375, MSC and ECFC cells evaluated by RT-PCR analysis in basal conditions (panel A), in A375 cells incubated with CM-MSC and with CM-ECFC (panel B). Panel C: SDF-1 expression after the knockdown of SDF-1 gene in MSC. Panel D: ECFC Matrigel invasion by adding in the lower compartment of Boyden chamber of: control media (picture 1); conditioned media from A375 (picture 2); CM-MSC (picture 3), and CM from cocolture A375/MSC (picture 5). Picture 4 and 6 show ECFC invasion upon addition in the lower compartment of CM from SDF1-silenced MSC or from A375/MSC SDF1-silenced cocolture, respectively; picture 7 and 8 show the invasion of ECFC in the presence of anti-CXCR4 antibody, in control condition (picure 7) and after CM-co-colture incubation (picture 8). Histogram on the right represent the quantification of Matrigel invasion performed by counting migrated cells as described in Figures 1A. *Asterisks* (* *P*<0.05) indicate significant difference between the indicated experimental condition. Panel E: *in vivo* experiments. A375 melanoma cells were injected alone (control) or together with MSC in CD1-nude mice and the development of tumor was followed by measuring the tumor volume. Five mice were used for each experimental condition and the results are the mean of 3 different experiments performed in triplicate. *Asterisks* indicate significant difference (* *P* < 0.05) from control. Panel F: *In vivo* recruitment of ECFCs into tumor mass. CD1-nude mice were grafted subcutaneously with viable melanoma cells together with MSC and then ECFCs, radiolabelled with ^111^In 8-oxyquinoline (oxine), were injected intravenously when the tumor reached about 150 mm^3^ volume, in the absence or in the presence of anti-CXCR4 or irrelevant antibody. 24 hours after radiolabelled ECFCs administration, SPECT tomography was performed as described in material and method. SPECT images show a selective visualization of radioactive ECFCs in the tumor mass. Figure shows a representative experiment (n=3). On the left: SPECT images of ECFC engraftment in the tumor mass at 30 min and 24 h (upper panel) and ECFC engraftment in the presence of irrelevant IgG or anti-CXCR4 antibody (lower panel). On the right: relative histogram of uptake efficiency.

Recruitment of EPCs and MSCs in neoplastic tissues is a complex process primarily regulated by the interaction of various chemokines with their receptors. We evaluated the expression of SDF1/CXCR4 system in MSCs, ECFCs and tumor cells, as shown in Fig 4. PCR analysis revealed that MSCs expressed high level of SDF1, ECFCs expressed the related CXCR4 receptor, while A375 cells expressed very low levels of both CXCR4 and SDF1 genes (Fig.4A). Incubation of A375 with CM-MSC or CM-ECFC up-regulated the expression of SDF1 and its receptor (Fig. 4B). These results prompted us to use: i) MSCs as tumoral promoters by co-injecting them in mice together with melanoma cells and ii) ECFCs as carriers of MMP12, which can be recruited within tumors following the chemotactic gradient of SDF1 expressed by MSCs, normally present in tumors, but also able to up-regulate SDF1 expression in tumor cells [35]. Before *in vivo* experiments, we evaluated whether the SDF1/CXCR4-dependent ECFCs recruitment into tumor mass could be an experimentally supported possibility, by performing ECFC in vitro chemoinvasion assay upon addition of CMs in the lower well of the migration chamber. CMs from A375 (CM-A375), from MSCs (CM-MSC) and from a coculture of mixed (2:1) A375 and MSCs (CM-A375/MSC) were prepared by maintaining the cells 24h in colture medium. As shown in Fig.4D, CM-A375 induced a small increase of ECFCs Matrigel invasion, while CM-MSC were more effective. This effect was further strengthened by CM of A375/MSC co-cultures. To elucidate the involvement of SDF1/CXCR4 system, we performed *in vitro* Matrigel invasion of ECFCs after SDF1 siRNA treatment of MSCs (Fig.4C) or in the presence of CXCR4 blocking monoclonal antibody, taking in account for the toxic effect of CXCR4 siRNA. As a result of both treatments, ECFCs invasion was strongly impaired, thus indicating that ECFCs were recruited *in vitro* by SDF1 released by MSCs and also by cancer cells, as demonstrated by the inhibition of CXCR4/SDF1 in the co-colture experiments (Fig.4D). On the basis of these results, we investigated the *in vivo* role of MSC on tumor growth by injecting 1×10^6^ viable A375 cells alone or together with 0.5x10^6^ MSCs into CD-1 nude (nu/nu) mice, 6 to 8 weeks old (as described in Materials and Methods). Tumor development was monitored at regular intervals by measuring tumor diameters. We observed a drastic increase of tumor growth in the presence of MSCs co-injected with tumor cells (Fig. 4E) thus confirming a protumoral role of MSCs.

### The *in vivo* recruitment of ECFCs into tumor mass depends on SDF1-CXCR4 interaction

To evaluate ECFCs homing into tumor mass *in vivo*, 25 days after tumor cell implantation, when tumor reached about 120 mm^3^ volume, mice were subjected to intravenous injection of 2x10^6^ ECFCs labelled with 60 μCi of ^111^In 8-oxyquinoline (oxine). After 24 hours from administration, the scintigraphic imagine showed that radiolabeled ECFCs were localized only in the tumor mass (Fig 4F). To evaluate the involvement of CXCR4 in the ECFCs recruitment, we performed the aforementioned *in vivo* experiment in the presence of anti-CXCR4 antibody to block the receptor on ECFCs cell membrane surface. In this condition we observed the failure of ECFCs to home into tumor mass, confirming the involvment of the SDF1/CXCR4 system in the *in vivo* recruitment of ECFCs (Fig. 4F). Homing efficiency has been evaluated using the analysis software Vivid as described in material and methods and expressed as uptake efficiency. We found that ECFC intratumoral engraftment in control mice was 1.85% ± 0.2 compared to the muscle tissue controlateral while, in the presence of anti-CXCR4 antibody, the uptake was significantly reduced to 0.6% ± 0.1 (Fig. 4F).

### Construction of LV vector encoding MMP12. Characterization and activity of engineered ECFCs

**Figure 5:**
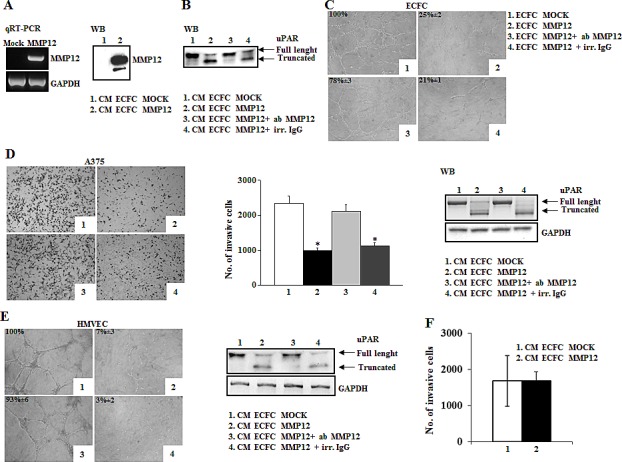
The anti-tumoral efficacy of MMP12-engineered ECFC *in vitro* and *in vivo* Panel A: Characterization of MMP12-engineered ECFC. RT-PCR shows MMP12 expression of engineered ECFC and Western blotting analysis detects MMP12 released in conditioned medium of ECFC containing empty vector (CM-ECFC MOCK, lane 1) or containing lentiviral vector encoding MMP12 molecule (CM-ECFC MMP12, lane 2). Panel B: Western blotting analysis of standard uPAR (st) incubated with aliquots of CM-ECFC MOCK (lane 1) and of CM-ECFC MMP12 (lane 2) in the presence of anti-MMP12 antibody (lane 3) or an irrelevant IgG (lane 4). Panel C: Capillary morphogenesis at 6h of ECFC-MOCK (picture1) and ECFC-MMP12 (Figure 2), in the presence of anti-MMP12 antibody (picture 3) or irrelevant IgG (picture 4). Numbers: percent field occupancy, taking control as 100%. Data are from 3 experiments performed in triplicate. Panel D: Anti-tumor property of ECFC-MMP12. Matrigel invasion of A375 cells in control conditions (picture 1) and upon addition, in the lower well of the migration chamber, of CM ECFC-MMP12 (picture 2), in the presence of anti-MMP12 antibody (picture 3) or irrelevant IgG (picture 4). The histogram represents the quantification of Matrigel invasion experiments, evaluated as in Fig.1A. *Asterisks* (*: *P*<0.05) indicate significant difference between the indicated experimental condition. Full length and truncated form of uPAR in lysates of A375 melanoma cells, shown on the right, was evaluated by Western blotting in the same conditions described above. Data result from three independent experiments. Panel E: Anti-angiogenic property of ECFC-MMP12. Capillary morphogenesis of endothelial cells treated with CM-ECFC MOCK (picture 1) or CM-ECFC MMP12 (picture 2) in the presence of anti-MMP12 antibody (picture 3) or irrelevant IgG (picture 4). Numbers: percent field occupancy, taking control as 100%. The pictures are representative of 3 different experiments performed in triplicate that gave similar results. Full length and truncated form of uPAR in lysates of endothelial cells, shown on the right, was evaluated by Western blotting in the same conditions described above. Panel F: Histogram represents number of A375 cells allowed to invade across the Matrigel pre-incubated overnight with CM ECFC MOCK and CM ECFC MMP12.

In order to deliver the anti-tumoral MMP12 into the tumor mass, we produced a lentivirus encoding MMP12 and then we infected commandos ECFCs. We obtained engineered ECFCs able to release the relevant molecule (ECFC-MMP12) and ECFCs containing empty vector (ECFC-MOCK). Occurred infection was evaluated by PCR analysis of lysates and by Western blotting of the medium. As shown in Fig.5A ECFC-MMP12 expressed higher levels of MMP12 when compared to ECFC-MOCK, both at gene and protein level. To study the enzymatic activity of the released MMP12, we incubated standard uPAR with CM ECFC-MMP12 or CM ECFC-MOCK in the presence or absence of anti-MMP12 antibody and then we evaluated full-length and truncated uPAR by Western blotting analysis with anti-uPAR R4 antibody. CM ECFC-MOCK did not cleave uPAR, whereas CM ECFC-MMP12 produced truncated uPAR (Fig 5B). Such truncation was inhibited by anti-MMP12 antibody, but not by irrelevant IgG. Moreover, MMP12 over-expression prevented ECFCs capillary morphogenesis in a MMP12-dependent manner, since this effect was reverted by anti-MMP12 antibody, while irrelevant IgG was ineffective (Fig.5C).

To evaluate the potential of MMP12 gene-modified ECFCs as regard to uPAR-dependent tumor invasion and angiogenesis we performed A375 Matrigel invasion and capillary morphogenesis of control human microvascular endothelial cells (HMVEC) in the presence of CM ECFC-MMP12 or CM ECFC-MOCK. As shown in Fig.5D and, CM-ECFC-MMP12 impaired the invasion of A375 as well as the capillary morphogenesis of HMVEC when compared to CM-ECFC-MOCK, confirming a MMP12-dependent anti-tumor and anti-angiogenic activity, as it was reverted by anti-MMP12 antibody. In these experiments we also demonstrated that the MMP12-dependent anti-tumor and anti-angiogenetic activity were related to the presence of the truncated form of uPAR. In fact, the uPAR cleavage was reversed in the presence of anti-MMP12 antibody (fig 5 D, on the right and E, on the right).

As it is well known that MMP12 is able to cleave a number of other substrates, we evaluated this possibility in our experimental conditions. Some authors ascribed the anti-angiogenic and anti-tumoral role of MMP12 to the production of angiostatin from plasminogen. We did not reveal any angiostatin production (data not shown). To evaluate whether the invasion property of A375 in the presence of CM-MMP12 was due to direct effects of exogenously supplied MMP12 on Matrigel other than uPAR cleavage, we incubated overnight the Matrigel coated filter with CM ECFC-MOCK or CM ECFC-MMP12 and then performed invasion assay of control A375 cells. Such a treatment did not change the A375 invasion properties (fig 5F). These results suggested that, in our experimental conditions, MMP12-mediated uPAR cleavage was required to inhibit A375 invasive properties.

### Effect of MMP12- engineered ECFCs on tumor growth and metastasis *in viv*o

**Figure 6:**
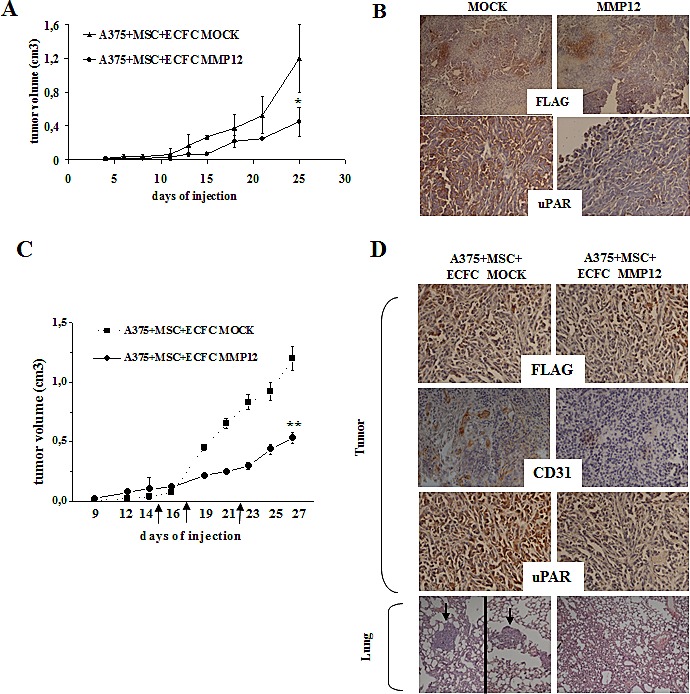
The *in vivo* effect of MMP12-engineered ECFC on tumor growth and metastasis Panel A. Melanoma tumors were obtained by subcutaneous injection of 1×10^6^ viable A375 cells together with 0.5× 10^6^ MSCs in the flanks of 6-week-old nude nu/nu (CD-1) and, when tumor mass was evident, mice were treated by i.v. injection with ECFC-MOCK or ECFC-MMP12, as described in materials and methods. The days of injection are depicted on the chart using big arrows. Effect of ECFC-MMP12 on *in vivo* tumor growth. Mixture of A375 melanoma cells and MSC were co-injected together with ECFC-MOCK or ECFC-MMP12 in nude mice. Tumor development was monitored for 25 days, by measuring tumor diameter, and then mice were sacrificed. *Asterisks indicate* significant difference (*: *P* < 0.05) from control. Panel B: uPAR and Flag levels assessed by immunohistochemistry in tumor tissue from mice treated as indicated in A and collected at the end of the experiment, as described in results section. These results are representative of 3 different experiments performed in triplicate that gave similar results (magnification: × 100 in the upper panels and × 400 in the lower panels). Panel C: Tumor growth curve was obtained by measuring tumor diameters at regular intervals. Eight mice were used for each experimental condition Statistical analysis was carried out by Student's t-test and significant differences between the two groups were indicated by the *asterisks* (**: *P* < 0.001 from control ECFC-MOCK). Panel D: Histological analysis of the tumors and lungs recovered at autopsy as described in the text. Tumor slides were performed according to standard procedures and incubated with a primary antibody against CD31, uPAR and FLAG (see Materials and Methods) followed by a peroxidase-conjugated IgG preparation; 3,39-diaminobenzidine was used as the chromogen for development. Slides werecounterstained with aqueous Meyer hematoxylin, mounted with glycerol for visual inspection and esamined under bright field microscope. Lungs were stained with hematoxylin–eosin. Metastases (depicted using big arrows) in the image of mouse injected with ECFC MOCK come from two different slides of the same lung. Pictures are representative of four randomly chosen microscopic fields (magnification: × 400).

With the purpose to evaluate the therapeutic potential of ECFC-MMP12 for melanoma growth in vivo, a mixture of 1×10^6^ viable A375 and 0.5x10^6^ MSCs were co-injected into 6 to 8 weeks old CD-1 nude (nu/nu) mice together with 0.5x10^6^ ECFC-MMP12 or ECFC-MOCK. In the presence of ECFC-MMP12 tumor growth was strongly inhibited: after 25 days the volume of tumors containing ECFC-MM12 was 0.4±0.15 cm^3^ compared to 1.2±0.2 cm^3^ tumor containing ECFC-MOCK (Fig 6A). The presence of viable ECFCs as well as the presence of full-length uPAR were evaluated in recovered tumor mass by immunohistochemical analysis (Fig 6B). On the basis of the use of FLAG expression vectors in the construction of lentivirus, the presence of ECFCs was estimated by using an anti-FLAG antibody while the efficiency of uPAR cleavage was evaluated by a mAb which recognizes only the full-length native form (uPAR N-19, Santa Cruz). Anti-FLAG antibody stained tumors explanted from mice injected with ECFC-MMP12 as well as with ECFC-MOCK, thus demonstrating that engineered ECFCs remained within tumor mass. The anti-uPAR N-19 antibody stained only tumor cells of ECFC-MOCK- treated mice, while the staining was strongly reduced in tumor mass of mice treated with ECFC-MMP12 (fig. 6B). These results demonstrated the MMP12-dependent cleavage of full length uPAR, which is in agreement with the inhibition of tumor growth by ECFC-MMP12 (Fig. 6A).

To evaluate the effect of MMP12-dependent uPAR cleavage on tumor growth and on metastasis development in tumor bearing mice, we injected ECFC-MMP12 or ECFC-MOCK when tumor mass was evident in all the mice injected with A375 cells and MSC. We observed a significant decrease of tumor growth in mice which received i.v. ECFC-MMP12, while in mice treated with ECFC-MOCK the tumor continued to grow (Fig. 6C). At the end of treatment tumor mass and lung were subjected to histological analysis (Fig 6D). We demonstrated the presence of injected ECFCs in tumor mass of all the mice by using an anti-FLAG antibody. We also examined the presence of full-lenght uPAR using the anti-uPAR N-19 antibody and angiogenesis by using anti-CD31 antibody. Both antibodies stained only tumor cells of ECFC-MOCK-treated mice, while the staining was strongly decreased in tumor mass of mice treated with ECFC-MMP12. These results are indicative of a MMP12-dependent cleavage of full length uPAR and the intratumoral angiogenesis inhibition, which are in agreement with the inhibition of tumor growth. We also examined the lungs to evaluate the presence of metastasis. The ECFC-MMP12 treated mice did not show any metastasis, while the control mice exhibited a mean of 5-6 metastasis/lung, thus demonstrating an anti-metastatic effect of injected ECFC-MMP12.

**Figure 7:**
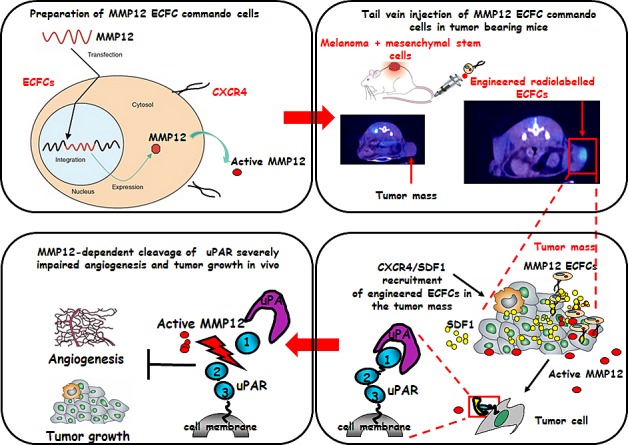
Schematic representation of the concept of ECFCs as cellular vehicles delivering anti-tumor uPAR degrading enzyme ECFCs derived from cord blood, were transduced with lentiviral vectors encoding the matrix metalloproteinase 12 (MMP12). Subcutaneous tumors were developed by direct co-injection of A375 and MSCs. Six days after tumor cells implantation, genetically modified ECFCs labelled with 60 μCi of 111In 8-oxyquinoline (oxine) were injected intravenously into CD-1 nude (nu/nu). 24 hours later the scintigraphic imagine showed that radiolabeled ECFCs were localized only in the tumor mass. High expression of SDF1 by tumour cells and MSCs form a local gradient of the chemokine in the tumour region. CXCR4-expressing ECFCs are thus recruited to the tumour, where they release active MMP12 enzyme which cleaves uPAR impairing angiogenesis and tumor growth.

In fig. 7 is shown a schematic representation of our results: ECFC-MMP12 recruited in tumor mass, by exploiting the CXCR4/SDF1 system, released the MMP12 anti-tumor uPAR degrading enzyme, thus impairing tumor growth and angiogenesis.

## DISCUSSION

Tumor progression is characterized by increased expression of uPA/uPAR system [7]. This system is finely modulated: it is transiently expressed whenever cell movement is required, as in inflammation, wound repair and tumors, while its expression is low in normal quiescent tissues. In the tumor microenvironment uPAR is expressed not only in tumor cells but also in endothelial cells (EC), fibroblasts, inflammatory cells (macrophages, neutrophils) and in MSC thus becoming an attractive target for cancer therapy [36, 37]. Our previous studies [8, 10]showed that movement of endothelial and cancer cells depends on the native full-lenght uPAR, and that truncation of uPAR domain 1 by MMP12 results into the loss of their invasion properties. In the last years a new idea about the role of MMPs in cancer is emerging. Several MMPs, including MMP12, display a protective effect by suppressing tumor growth, thus accounting for the failure of clinical trials which employed broad-range MMP inhibitors [17, 20].

Several strategies are emerging to target uPAR [38, 40]. A new anti-cancer therapeutical strategy is the “targeted therapy”, as opposed to traditional nonspecific chemotherapy, thus reducing pharmacological side effects and increasing the efficiency of the therapeutic approach. Due to its activity, MMP12 can be considered a biological drug to be used in targeted anti-uPAR therapy. To deliver such molecules the so-called “cell-based therapies” are becoming more relevant. Due to their capability to home within tumors, MSC and EPCs are considered the best candidates to be used as “Trojan Horses” carrying anti-cancer drugs [41-43].

EPCs play an important role in tumor vascularization and metastatic transition either by their incorporation in vessels or by supporting tumor angiogenesis via paracrine secretion of proangiogenic cytokines. EPCs are selectively recruited within the tumor mass [41]. The amount of circulating EPCs positively correlates with tumor progression and their levels decrease after chemotherapy [26]. Mesenchymal stem cells (MSCs) are involved in tumor growth by providing a stromal support and by secreting angiogenic factors. Subcutaneous co-injection of MSC and tumor cells exalts tumor progression [27, 28]. Moreover, over-expression of uPA-uPAR in tumors plays an important role in recruitment of MSC in malignant solid tumors [44].

Here we manipulated ECFCs, a sub-type of EPCs, as cellular vehicle to deliver MMP12 into the tumor mass with the aim to truncate uPAR both in endothelial and tumor cells. We chose the solid tumor melanoma because its distinctive features: it often affects patients < 60 years, its prognosis is mostly related to the thickness of primary tumor in the skin and in its late phases (stage IV) it is poorly responsive to conventional treatments. We hypothesized that delivery of ECFCs engineered to express MMP12 into human melanomas transplanted in nude mice could release such metalloproteinase into tumor mass thereby controlling melanoma progression.

In this study we first confirmed that uPAR levels correlated with tumor malignancy in different melanoma cell lines and then we evaluated the role of microenvironment on the invasive properties of melanoma cells. We demonstrated that incubation of melanoma cells with CM-ECFC or CM-MSC increased the invasion of tumor cells, which depends on the up-regulation of full length uPAR. This treatment also induced the down-regulation of MMP12, which is in agreement with full length uPAR-dependent tumor malignancy, thus confirming a “protective role” of MMP12 in cancer invasivity. To further verificate the role of MMP-12 in inhibiting tumor invasion, we transiently transfected A375 melanoma with MMP12 expression vector and demonstrated that its consequent upregulation impaired uPAR-dependent melanoma cell invasion.

It is known that EPCs recruitment into tumoral tissues depends on the interaction of tumor chemokines with related receptors expressed on EPCs. In particular, CXCR4/SDF1 system plays a pivotal role in EPCs tumor homing [45-48]. Herein, we demonstrated that ECFCs express CXCR4 on their surface, while MSCs secrete large amounts of SDF1. Moreover, melanoma cell incubation with CM-ECFC or CM-MSC greatly up-regulated this system. We have also shown that the *in vitro* invasivity of ECFCs and *in vivo* ECFCs tumor homing depend on the interaction between CXCR4 of ECFCs and SDF1 released by MSCs and melanoma cells.

On the basis of these results, we used MSCs as tumoral promoters by co-injecting them in mice together with melanoma cells, with the aim of enriching tumor microenvironment with SDF-1 molecular magnets able to recruit ECFC-MMP12.

We engineered ECFCs with a lentivirus encoding the uPAR-degrading enzyme MMP12 in order to induce a lentivirus-dependent “gain of function” of MMP12 activity in ECFCs. This ex vivo manipulated ECFCs lost the capacity to perform capillary morphogenesis *in vitro* and, at the same time, acquired the anti-tumor and anti-angiogenetic activity thus impairing invasion of melanoma cells and the capillary morphogenesis of control ECs. *In vivo* co-injection of melanoma cells with ECFC-MMP12 strongly inhibited tumor growth and, at the same time, cleaved uPAR. These results are strongly suggestive of an uPAR-dependent inhibition of tumor angiogenesis and tumor spread. Finally, the i.v injection of ECFC-MMP12s in melanoma bearing mice induced a significant decrease of melanoma growth and inhibited the development of lung metastasis. Immunohistochemical analysis of tumor mass revealed that the delivered ECFCs remained viable within implanted melanomas. Simultaneously, we observed a lower amount of full length uPAR and inhibition of angiogenesis. While confirming the data obtained *in vitro* these results also indicate that the release of MMP12 by engineered ECFCs recruited in the tumor mass impairs tumor growth and metastasis mainly by inhibiting uPAR-dependent angiogenesis and spread of melanoma cells.

Many viral constructs have been designed to deliver new therapeutic information into cancer cells, but none of such viruses include an MMP12 construct. In this study we suggest, for the first time, the employment of MMP12 as biological drug in the anti-tumoral cell-based therapy in order to impair tumor angiogenesis and tumor invasion of melanoma and of all those tumor which heavily depend on uPAR to perform invasion.

Knowledge sprouting from these results indicate the possibility to reduce tumour angiogenesis and tumor progression in melanoma-bearing patients through injection of autologous EPCs able to deliver the MMP12 anticancer agent into tumor mass, which could inhibit all the uPAR-dependent melanoma progression characteristics. EPCs are a minor component of circulating cells, but a series of methods are available for their separation and ex-vivo expansion from human blood after a proper stimulation of bone marrow [49, 50]. The use of autologous ECFCs represent an effective melanoma tumor treatment without rejection risk, that may cooperate with standard therapy.

Results of this research have a translational relevance and a potential impact for the health system related to a better control of metastatic disease in melanoma. An approach based on i.v. injection of MMP12-engineered ECFCs, able to impair tumor progression (therapy), and on the labelling of ECFC with ^111^In-oxine for molecular imaging of primary and metastatic tumor masses (diagnosis), provides a new and promising MMP12 theranostic approach to human melanoma and to future cancer medicine for all the tumor displaying uPAR-dependent cancer progression.

## MATERIAL AND METHODS

### Cell lines

Endothelial Colony-Forming-Cells (ECFCs), a subpopulation of EPCs, were isolated from >50 ml human umbilical cord blood (UCB) of health newborns, as described [10], after maternal informed consent and in compliance with Italian legislation, and analyzed for the expression of surface antigens (CD45, CD34, CD31, CD105, ULEX, vWF, KDR, uPAR) by flow-cytometry [10]. Human microvascular endothelial cells (HMVEC) are available in our laboratory. Mesenchymal Stem Cells (MSCs) were obtained from bone marrow aspirates of donors which signed informed consent, and were expanded according to published methods (29). MSC were analyzed at P0 and P12 for the expression of surface antigens CD45, CD14, CD44, CD166, CD90, CD73, HLA-DP, -DQ, -DR, HLA-ABC, CD105, CD271 APC (FACSCalibur, Becton Dickinson). At P0 and P6 an aliquot of cells was used to test the adipo- and osteo-differentiation capabilities. The melanoma cell lines A375 and Mewo were obtained from American Type Culture Collection (Manassas, VA) and grown in Dulbecco's modified Eagle's medium (DMEM) with 10% FBS (Euroclone). M14 melanoma cells were available in our laboratory.

### Preparation of conditioned medium (CM-ECFC and CM-MSC)

ECFC and MSC cells were seeded at density of 2.0 × 10^5^ cells/ml in complete media into T-75 tissue culture flasks and grown to approximately 80% confluency. Cells were collected washed twice with phosphate-buffered saline (PBS) and incubated overnight in 2% FCS medium. Overnight condition media were collected and centrifuged at 700 g at room temperature for 5 min to pellet cells. The supernatant was collected and centrifuged at 2000 g at 4°C for 10 min to remove cell debris. The supernatant representing ECFC-CM and MSC-CM was divided into two aliquots one subjected for MMP12 detection and the second aliquot was used for conditioning A375 cells. In some experiments, conditioned medium (CM) was prepared with MSCs pretreated with small-interfering-RNA (siRNA) and negative control constructs obtained from Riboxx Life Sciences/Euroclone (IBONI siRNA-pool, targeting human SDF1). siRNA constructs were incorporated into RiboxxFECT transfection reagent according to the manufacturer's instructions. Cells were incubated for 48 hours with transfection mix.

### Western blot analysis, semiquantitative and quantitative PCR

Aliquots of culture media (20 μl) and lysates (40μg) of A375 incubated for 24 hours with CM-ECFC or CM-MSC, were subjected to Western blotting. The primary antibodies were: anti-uPAR R3 (1:1000; mouse monoclonal antibody, BioPorto Diagnostics), which recognizes the full-length uPAR; anti-uPAR R4 (1:1000 mouse monoclonal antibody, BioPorto Diagnostics) which reveals both the full-length and truncated form of uPAR; anti-MMP12 (1:1000; rabbit polyclonal antibody, Millipore) and anti-GAPDH (mAbcam 9484) as loading control. After incubation with horseradish peroxidase–conjugated donkey anti-mouse or anti-rabbit or anti-goat IgG (1:10,000) for 1 hour, immune complexes were detected with an enhanced chemiluminescence ECL detection system (GE Healthcare). The membranes were exposed to autoradiographic films for 1–10 minutes.

Total RNA was prepared using Nucleospin RNA II (Macherey-Nagel), agarose gel checked for integrity, and reverse transcribed with GoScript system (Promega) using random primers according to manufacturer's instructions. Selected genes were evaluated, before and after CM-ECFC and CM-MSC treatment, by a quantitative Real-Time (RT)–PCR with 7500 Fast Real Time PCR System (Applied Biosystems) and determined by the comparative C_t_ method using 18S ribosomal RNA as the normalization gene. Amplification was performed with the default PCR setting: 40 cycles of 95°C for 15 seconds and of 60°C for 60 seconds using SYBR Green–based detection (GoTaq qPCR Master Mix; Promega). mRNA levels of uPAR, CXCR4, MMP12 and SDF-1 were determined by an internal-based semiquantitative RT–PCR, (MJ Research) using procedures previously described [10, 30]. GAPDH was used as loading control. The reaction products were analyzed by electrophoresis in 2% agarose gel containing ethidium bromide. Primer sequences (IDT, TemaRicerca) are reported in Table 1.

**Table 1:** 

Gene	Forward primer	Reverse primer
MMP-12	5'-tgctgatgacatacgtggca-3'	5'-aggatttggcaagcgttgg-3'
MMP-12	5'-cctgctttgtcctttgatgc-3'	5'-tgactcaatccctggaaagtc-3'
CXCR4	5'-gccttatcctgcctggtattgtc-3	5'-gcgaagaaagccaggatgaggat-3'
uPAR	5'-gcccaatcctggagcttga-3'	5'-tccccttgcagctgtaacact-3'
SDF1	5'-actgggtttgtgattgcctctgaag-3'	5'-ggaacctgaacccctgctgtg-3'
18s rRNA	5'-cggctaccacatccaaggaa - 3'	5'-gctggaattaccgcggct -3'
GAPDH	5'-ccacccatggcaaattccatggca-3'	5'-tctagacggcaggtcaggtccacc-3'

### Invasion assay and capillary morphogenesis

Cells invasion was studied in Boyden chambers in which the upper and lower wells were separated by 8 μm–pore size polycarbonate filters coated with Matrigel (50 μg/filter; BD Biosciences). Cells (15×10^3^) were placed in the upper well, and invasion was performed for 6 hours at 37°C-5% CO_2_ as previously described [8, 10]. A375 invasion was also evaluated either by adding CM-ECFC or CM-MSC in lower well or after 24 hours preincubation with DMEM–2% FCS, or with CMs-2% FCS. In some experiments, invasion was performed in the presence of 2 μg/ml uPAR-neutralizing antibody R3 or anti-MMP12 polyclonal antibodies, or anti CXCR4 (R&D Systems) or irrelevant IgG (Sigma). Some invasion experiments were performed with the A375 transiently trasfected with MMP12. In some chemoinvasion experiment the filters coated with Matrigel were pre-incubated overnight with CM ECFC MOCK and CM ECFC MMP12. The day after the CM removed and untreated A375 allowed to invade out of the matrix, across the membrane.

In vitro capillary morphogenesis was performed as described [10] in tissue culture wells coated with Matrigel. ECFCs or HMVECs were plated (18x10^3^/well) in EBM-2 medium, supplemented with 2% FCS and incubated at 37°C-5% CO2, under condition described in Fig 5. Results were quantified at 6h by measuring the percent field occupancy of capillary projections. Six to nine photographic fields from three plates were scanned for each point. Results were expressed as percent field occupancy ± SD with respect to control fixed at 100%.

### MMP12 transient transfection and uPAR cleavage

The cDNA-containing vector and the empty plasmid (15 μg each of either *pCDNA3.1* + *MMP12* or *pCDNA3.1* alone; Invitrogen) were transiently transfected into A375 cells using Lipofectamine Tranfection Reagents (Invitrogen Co.)[10]. Briefly, 3.5×10^5^ cells/well were plated in six multiwell plates 24 hrs before transfection and then complete medium was replaced. Expression of *MMP12* gene was measured by Real Time-PCR and the level of released protein was analyzed by Western blotting of culture medium.

To verify MMP12-dependent uPAR cleavage, 25 μg of recombinant human uPAR (R&D Systems) was incubated overnight at 37°C with 15 μl of DMEM–2% FBS or CM derived from empty vector or *MMP-12* transiently transfected A375 or with CM derived from ECFCs engineered to express MMP12. These experiments were performed in the absence and in the presence of anti-MMP12 antibody (10 μg/ml), as described [10].

### SDS-PAGE Zymography

MMP12 α-elastin-degrading activitiy was examined by electrophoresis of the culture medium under non reducing conditions in 12.5% polyacrylamide gels containing α-elastin (1.2 mg/ml) (Sigma Chemical Co.) [31]. After electrophoresis, the gels were washed in 2.5% Triton X-100 for 30 minutes and then incubated overnight at 37°C in elastase assay buffer. Lysis of the substrate in the gel was visualized by staining with Coomassie R-250 (0.5% [wt/vol]), followed by destaining.

### Lentiviral vector construction, production and cell transduction

To construct LvPGK-MMP12Flag, the MMP12-Flag DNA sequence was amplified from the pCDNA3 plasmid using primers containing BamHI and BsrGI restriction sites (BamHI-5'cgggatcccgatggggaagtttcttc-3' and BsrGI 5'ctgctactgctatttatcgcacatgtgc-3') and subsequently cloned in the lentiviral vector pRRLsin.PPT.hPGK.mcherry_pre (kindly provided by dr Marco Erreni, Humanitas Clinical and Research Center, Milan, Italy), replacing the mcherry sequence. Similarly, the LvPGK-Flag control vector was generated by synthesizing the Flag-tag sequence at 95°C for 1 hour, using primers containing BamHI, ClaI and BsrGI restriction sites (5'-cggga-tcccgtactacaaagacgatgacgataaatagccatcgatggt-3' and 5'-cgtgtacaccatcgatggctatttatcgtcatcgtctttgtag-tacgggatcccg-3'). All amplification conditions were settled using the Phusion Hot Start II high-fidelity DNA Polymerase (Finnzymes), according to manufacturer's instructions. Sequence integrity was verified by standard sequence analysis (Primm srl, Milan, Italy). The third-generation lentiviral vectors were produced by transient transfection of 293T cells according to standard protocols [32]. Briefly, subconfluent 293T-cells were co-transfected with 11,7 μg of the transfer plasmid, 5 μg of packaging plasmid, 3.5 μg of envelope plasmid and 5 μg of rev-expressing plasmid (ViraPower™ lentiviral packaging mix, Invitrogen) by Lipofectamine (Invitrogen). After 16 h medium was changed, and 24 h later recombinant lentivirus vectors were collected, filtered through 0.22-μm-pore-size cellulose acetate filters and immediately used. To determine the maximum viral efficiency, serial dilutions of freshly harvested conditioned medium were used to infect 10^5^ ECFC in a six-well plate in the presence of Polybrene (8 μg/ml). After 24 hours ECFC were incubated with complete medium, EBM-2 containing EGM-2 Single Quots (Lonza), and 48 h later analyzed for infection efficiency, by Western blot.

### *In vivo* experiments

All procedures involving animals were performed in accordance with the ethical standards and according to the Declaration of Helsinki and according to national guidelines, approved by the ethical committee of Animal Welfare Office of Italian Work Ministry and conformed to the legal mandates and Italian guidelines for the care and maintenance of laboratory animals.

To evaluate the effect of MSCs on tumor growth, five male CD1 immunodeficient mice (6–8 weeks old; Charles River Laboratories International) were injected subcutaneously with 100 μl of PBS containing 1x10^6^ A375 cells alone and five mice with A375 cells together with 0.5×10^6^ MSCs. Tumor development was monitored at regular intervals by measuring tumor diameters by a calliper. Mice were sacrificed 25 days after tumor cell implantation and tumor growth was estimated by measuring the tumor volume determined by the formula: (L × W ^2^)/2, where L and W are the length and width.

For ECFCs tumor homing, ECFCs were resuspended in medium containing 100 μCi of 111In 8-oxyquinoline (oxine) (Perkin Elmer)/10^6^ cells. After washing, 2−3×10^6^ ^111^In 8-oxyquinoline (oxine) labeled cells were infused intravenously into 12 tumor bearing nude mice. Six tumor bearing mice were injected with labelled ECFCs pre-incubated with anti-CXCR4 antibody (R&D Systems, Minneapolis, MN; 10 μg/ml) and six mice were injected with labelled ECFCs pre-incubated with irrelevant IgG for 30 min. Animals were subjected to SPECT scan using the microPET/SPECT/CT Trifoil Imaging [33]. Postlabeling viability exceeded 80%, and preliminary experiments demonstrated adequate cell tracking. CT scan was performed followed by the SPECT tomography, starting at 30 min p.i. The CT/SPECT scan were acquired as previously described [34]. The energy window was set on the ^111^In photopeak ranging from 171 keV±10% and at 245 keV±10%. Once the scanning session was complete, animals were recovered in a housing unit and monitored until awake. The fused images were visually analyzed to detect the engrafted tumor mass or normal tissue and regional SPECT activities were measured using the analysis software Vivid (Gamma Medica-Ideas, Northridge, CA) by delineating the region of interest (ROI) of the tissue in the fused CT images. A similar ROI was generated for the muscle tissue on the contralateral side of the tumor. Tumor uptake efficiency (nTA) as related to the ROI under evaluation, was obtained using the following formula: nTA = TA-BA/BA, where TA is tumor activity, BA is background activity measured in the muscle tissue contralateral to the tumor and normalized to injected activity. The same formula was used to evaluate the uptake efficiency of different organs.

Evaluation of the therapeutic local effect of ECFC-MMP12 on the tumor onset, were performed by co-injecting A375 cells and MSCs into 10 nude mice together with 0.5x10^6^ MOCK (5 mice) or ECFC-MMP12 (5 mice). The animals were monitored daily and were sacrificed after 18 days to evaluate tumor growth. The histological analysis of tumor mass was performed as described below.

To evaluate the ECFC-MMP12 effect on *in vivo* tumor progression, we injected melanoma cells with MSC into nude mice and, after fifteen days, when the tumor mass was evident, we performed i.v. injection of engineered 2 × 10^6^ ECFCs, according to the following schedule: 8 mice were treated with ECFC-MOCKs and 8 mice with ECFC-MMP12s. The treatment was repeated after three and six days and then tumor growth was followed by measuring the tumor volume for five additional days, before the tumour masses exceeded a size producing evident physical discomfort. Removed tumors and lungs were fixed overnight at 4°C in formalin (5% in PBS) for histological analyses performed on paraffin-embedded sections, using the avidin-biotin-peroxidase complex method and diaminobenzidine tetrahydrochloride chromogen kit (Dako LSAB2; Dako Corporation, Carpinteria, CA). The sections were probed with anti-uPAR (N19, Santa Cruz Biotechnology), anti-Flag (cell Signaling), anti CD31 (Thermo Scientific). Slides were counterstained with aqueous Meyer hematoxylin and mounted with glycerol for visual inspection and photography. To evaluate the metastatic potential of melanoma cells, the mice's lungs were removed and the number of metastasis were counted under a stereomicroscope.

### Statistics

Results are expressed as means ± SD. Multiple comparisons were performed by the Student test. Statistical significances were accepted at * p<0.05 and **p<0.001.

## Abbreviations

uPAR: urokinase-type Plasminogen Activator Receptor
MMP12: Matrix-metallo-proteinase-12
HMVEC: Human Microvascular Endothelial Cell
ECFC: Endothelial Colony-Forming-Cell
MSC: Mesenchymal Stem Cell
SDF1: Stromal cell-derived Factor-1
CXCR4: CXC Chemokine Receptor 4
SPECT: Single Photon Emission Computed Tomography
